# Role of Probiotic *Bacilli* in Developing Synbiotic Food: Challenges and Opportunities

**DOI:** 10.3389/fmicb.2021.638830

**Published:** 2021-04-12

**Authors:** Carolina Szlufman, Moshe Shemesh

**Affiliations:** Department of Food Science, Institute of Postharvest Technology and Food Sciences, Agricultural Research Organization, The Volcani Center, Rishon LeZion, Israel

**Keywords:** probiotics, *Bacillus subtilis*, LAB, beneficial biofilm, synbiotic food, probiotic *Bacilli*, dietary fibers, symbiotic encapsulation

## Abstract

The human body is inhabited by a vast diversity of probiotic microorganisms that could positively affect human physiology. Besides, prebiotic food substances may induce symbiotic relationship among probiotic species through the successful establishment of commensal microbiota, whose connections with the host are multifaceted and multidirectional. As deliberated throughout this review, prebiotic and synbiotic foods contain the capability to stimulate numerous health characteristics in host organisms through various means. Predominantly, the normal microbiota fosters the digestion of food and may boost the innate and adaptive immune system’s functionalities. Therefore, live probiotic bacteria, for instance, probiotic *Bacilli* obtained together with prebiotic food, can help stimulate healthiness in humans. Thus, we discuss how certain dietary fibers may preserve the probiotic efficacy by serving as the scaffold for probiotic *Bacilli* to colonize them through forming symbiotic interactions. The fibers can essentially promote protection by encapsulating probiotic *Bacilli* against various environmental and physical stresses that might kill the free-living bacterial cells. Besides, these fibers would serve as prebiotic substances that would eventually be utilized for the proliferation of probiotic cells. It is believed that applying this conceptual idea will provide a novel platform toward developing probiotic and synbiotic foods, as discussed in this review.

## Introduction

The human body is normally populated with an extensive assortment of microorganisms that may have a positive impact on human physiology and functions, such as the symbiotic relationship of probiotics along with prebiotics in the prevention of diseases (Hooper and Gordon, 2001; Lebeer et al., 2010; Davani-Davari et al., 2019). Historically, much before scientific research could examine the impacts of microbes on the internal human environment, many probiotic species were being used in dietary consumption for centuries. For example, they were used in the fermentation of dairy products, such as cheese and yogurt, as well as wine (Bokulich et al., 2015; Wolfe and Dutton, 2015). We now know that probiotics are live microorganisms, which reside in an organism and can contribute beneficially to the host’ health (Lebeer et al., 2010; Fukuda et al., 2011; Piewngam et al., 2018). The probiotic supplements field is continuously growing since evidence suggests gut microbiota’s essentiality in promoting body healthiness and well-being (Clemente et al., 2012; Wu and Wu, 2012; Dhar and Mohanty, 2020). Therefore, possible manipulations of the microbiome composition in the gastrointestinal tract (GIT) of a host organism, specifically through consuming probiotic food, become a potential remedy.

The molecular interactions of the host with the microbiota are complex, numerous, and multidirectional. For instance, the microbiota of the human GIT exists in a crosstalk that ranges between mutualism and pathogenicity, fostered by residential and ingested microorganisms. The normal microbiota contributes to proper food digestion as well as the optimal functioning of the immune system (Hooper and Gordon, 2001). The gut microbiota is supposed to significantly regulate the development and function of the innate and adaptive immune system (Negi et al., 2019a). The gut microbiota and immune homeostasis seem to have a back and forth relationship and are a subject of intense research in the field of infectious diseases. Also, gut microbiota-derived signals regulate the immune cells for pro and anti-inflammatory responses, thereby affecting the susceptibility to various diseases (Negi et al., 2019b).

**FIGURE 1:**
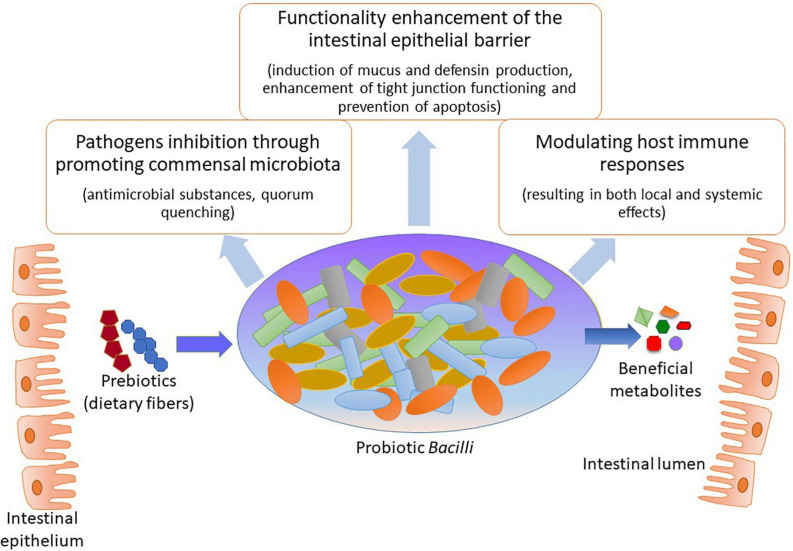
Health promoting modes of action by probiotic *Bacilli.*

Interest in the microbiota’s beneficial functions has resulted in the eventual selection of specific species with putative health-promoting capacities. To exemplify, the Gram-positive *Bacilli* are prominent colonizers of the human GIT and thus have been widely used as probiotic species in clinical applications (Lebeer et al., 2010; Ilinskaya et al., 2017). Among the main benefits of those species are the positive interactions with the host organism by metabolizing various dietary components that may affect commensal microbiota composition as well as inducing defense mechanisms against infectious diseases (Figure 1). Specifically, probiotic *Bacilli* can metabolize different prebiotic compounds that promote the healthiness of the host organism in which they reside using different mechanisms; these mechanisms include: pathogen obstruction due to antagonism and competition, pH level preservation, and defense of intestinal mucosal barrier and its functions (Ilinskaya et al., 2017; Davani-Davari et al., 2019; Seifert et al., 2019). In addition, probiotic *Bacilli* have been related to the production of many health-promoting factors for the host organism, for instance, vitamins and small molecules such as aminobutyric acid (GABA; Gu et al., 2015; Wang et al., 2019; Ren et al., 2020); this four-carbon non-proteinogenic amino acid is well-known for its diverse biological functions such as anxiety inhibition, sleep promotion, blood pressure reduction, diabetes treatment, and immune enhancement (Li et al., 2016; Wang et al., 2019).

Among the most crucial mode of actions of probiotic *Bacilli* in mitigating pathogenic species, from either intestinal or respiratory tract, appears to be by modifying the microbiota composition within the GIT through creating a more favorable balance in the microbial population (Fukuda et al., 2011; Gagliardi et al., 2018; Li et al., 2019). The exclusion of pathogenic species often occurs by two major mechanisms: (i) through production of antimicrobial substances that may eliminate the undesired bacteria (Caulier et al., 2019; Kimelman and Shemesh, 2019) and (ii) by affecting gene expression patterns of pathogenic microorganisms resulting in the suppressed ability to colonize the GIT of the host organism (Piewngam et al., 2018). Besides, probiotic *Bacilli* may affect both innate and adaptive immunity through upholding intestinal homeostasis as well as improving different aspects of GIT functionality (Fukuda et al., 2011; Lefevre et al., 2015; Jager et al., 2018; Johnson et al., 2019). By improving the digestibility of nutrients, for instance, certain types of indigestible dietary fibers, could probiotic *Bacilli* vastly contribute to the host organism’s healthiness (Rajasekharan et al., 2020). The pathogen eliminating, immunomodulatory and additional beneficial capabilities of probiotic *Bacilli* for the host organism, summarized in Table 1, paving the way for developing novel probiotic formulations as well as probiotic food for potential application in clinical dietetics as well as agriculture and food industry.

**TABLE 1:** Putative health-promoting modes of action by probiotic *Bacilli*.

**Mode of probiotic activity**	**Resulting functionality**	**Type of bacteria**	**References**
Modification of microbiota in the GIT	Creating a more favorable microbial population due to a shift in the balance toward beneficial microorganisms	Various probiotic *Bacilli*	Fukuda et al., 2011; Gagliardi et al., 2018; Vrancken et al., 2019
Alteration in gene expression of pathogenic microorganisms by affecting quorum sensing	Interfering signaling mechanisms in pathogenic bacteria through influencing their pathogenicity or survivability	*B. subtilis*	Piewngam et al., 2018
Production of antimicrobial substances: bacteriocins or lipopeptide compounds	Growth inhibition of pathogenic microorganisms in the intestine	Typical to all probiotic *Bacilli*	Hong et al., 2005; Caulier et al., 2019
Spore-forming capability	Remarkable ability to survive in harsh environmental conditions	Typical to all *Bacillus* species	Hong et al., 2005
Improving the digestibility of nutrients, mainly due to enhanced enzyme activity in the intestine, especially of α-amylase, cellulase, phytase, proteases, and metalloproteases	Optimize mineral absorption, carbohydrate digestion, reduction in cholesterol level, and production of nutrients.	*Bacillus coagulans*	Jager et al., 2018
Immunomodulation affecting both innate and adaptive immunity	Upholds intestinal homeostasis and improves adaptive immune response	*Lactobacillus* and *Bifidobacterium*	Fukuda et al., 2011; Yan and Polk, 2011
Protecting probiotic cells via inducing biofilm matrix production	Sustain enzymes and offer safety against osmotic stress, elevated temperatures, freeze thawing, and drying processing protocols	Different probiotic *Bacilli*	Yahav et al., 2018; Terpou et al., 2019
Production of health-promoting factors for the host organism, for instance, vitamins and small molecules such as (GABA)	Modulating diverse biological functions and immune enhancement of host organism	Different probiotic *Bacilli*	Li et al., 2016; Wang et al., 2019
Triggering anticariogenic activity	Effective metabolism of sugar alcohols reinforces the probiotic potential of *Bacillus* spp. against pathogenic *Streptococci*	*B. subtilis*	Duanis-Assaf et al., 2020
Inducing antiviral activity	modulating infectivity through either affecting microbiota composition or production antiviral substitutes	Different probiotic *Bacilli*	Lefevre et al., 2015; Johnson et al., 2019
Protecting against acute liver injury and hyperammonemia	Reducing inflammatory cell infiltration into the liver and decreasing ammonia levels	*L. salivarius*	Yang et al., 2020
Triggering anti-tumorogenic activity through producing a probiotic bacteriocin	Modulation of tumorogenic effect induced by periodontal pathogens	Different probiotic *Bacilli*	Kamarajan et al., 2020

## Current Challenges Toward Effective Applicability of Probiotic *Bacilli*

Preserving the efficacy of probiotic *Bacilli* exhibits paramount challenges that need to be addressed toward developing functional and health-promoting products, such as probiotic and symbiotic foods (Cruz et al., 2012; Yahav et al., 2018; Terpou et al., 2019). It was recently postulated that there could be a pronounced improvement in health when probiotics are administered along with antibiotics (Li et al., 2018). Moreover, probiotic organisms can comprise a solution to antibiotic resistance in certain conditions. Yet, there is a challenge due to broad-spectrum antibiotics usage, which could be targeting the beneficial probiotic bacteria too. On the other hand, complex microbial comminutes called biofilms have been revealed to stimulate antibiotic resistance; therefore, the biofilms could protect probiotic cells against the administered antibiotics. Besides, probiotic *Bacilli* are capable for the removal of pathogenic species such as *Staphylococcus aureus* from the intestinal and respiratory tract (Piewngam et al., 2018). This finding opens new thinking and opportunities for developing novel antimicrobial strategies instead of using standard or topical antibiotics.

Another challenge in the field is that the known prebiotic substances that can alter the gut microbiota do not include a protein source. Given that proteins digested in the small intestine provide a nitrogen source for commensal species, it is very limited and competitive among colonic bacteria (Seifert et al., 2019). Since some proteins possess functional attributes that make them suitable for the encapsulation of bioactive agents (Fathi et al., 2018), they may provide an excellent delivery system for the nanoencapsulation of appropriate probiotic species. This approach would further facilitate the development of protein-based symbiotic food.

### Dietary Fibers and Their Prebiotic Role

Dietary fibers, defined as carbohydrate polymers (which are neither digested nor absorbed), are normally subjected to bacterial fermentation in the GIT (Holscher, 2017); thus, they may impact the composition of bacterial communities as well as microbial metabolic activities, including the production of different fermentative end products (Hamaker and Tuncil, 2014; Bindels et al., 2015). Some dietary fibers can also be classified as prebiotic substances referred to as “selectively fermented ingredients that result in specific changes, in the composition or activity of the gastrointestinal microbiota, thus conferring benefit(s) upon host health” (Slavin, 2013; Holscher, 2017).

Studies have shown that the consumption of fibers in the diet may account for a decrease in mortality, coronary heart disease, cancer, type 2 diabetes, gastrointestinal issues, and strokes (Anderson et al., 2009; Reynolds et al., 2019). Blood pressure and cholesterol can also be decreased as a result of increasing fiber intake. Moreover, consumption of the fibers may improve conditions associated with glycemia and insulin issues in non-diabetic and diabetic persons. Additionally, it can enhance weight loss in obese individuals (Anderson et al., 2009).

It appears that fibers are nearly entirely broken down by the active microflora in either small or large bowel, mainly through fermentation processes (Anderson et al., 2009; Holscher, 2017). Therefore, dietary fibers fermented by the gut microbiota through producing certain metabolic substances can shape the immunological environment in the host organism and influence the severity of allergic inflammation (Trompette et al., 2014). Moreover, the normal gut microbiota can resist adipose tissue formation due to fiber consumption by the probiotic bacteria (Delzenne et al., 2019). Thus, fibers may significantly contribute to establishing and maintaining healthy gut microbiota, which would help against pathogens, expansion of the gut immune system, and synthesize health promoting metabolites (Hamaker and Tuncil, 2014; Reynolds et al., 2019).

Apparently, probiotic *Bacilli* consumption can also enhance the normal functionality of the GIT by reducing the inflammation rate in humans (Rhayat et al., 2019). Besides, it was shown that a *Lactobacillus reuteri* could reduce the blood cholesterol level in mice through increasing a ratio of high to low density lipoprotein, which might indirectly account for the permanency of the lactobacilli in the gut (Taranto et al., 2000). Despite many evidences for beneficial functionalities of bacteria and fungi in the GIT that can play a significant role in positive cross-talk with the host organism (Wolfe et al., 2014; Bokulich et al., 2015; Wolfe and Dutton, 2015), they have not been fully categorized or characterized up until now.

## Symbiotic Encapsulation as an Effective Method for Developing Future Probiotics

It is conceivable that using prebiotics to encapsulate and transport probiotics would result in the simultaneous distribution of pre and probiotics into the colon (Seifert et al., 2019). Accordingly, innovative encapsulation techniques have been suggested for the food and probiotics industry to shield probiotic species from severe storage environments and gastrointestinal conditions (Li et al., 2018). Another efficient method for coating beneficial microbes has been recently reported using biointerfacial supramolecular self-assembly of lipid membranes (Cao et al., 2019). This method exhibited significantly improved survival of bacterial cells against environmental assaults during oral delivery and treatment using two murine models of colitis (Cao et al., 2019). Besides, it was also reported that the integration of different factors as abiotic as well as biotic should be taken into account during proper selection method for probiotic encapsulation for the specific system (Šipailienë and Petraitytë, 2018).

Additional bio-coating technique established lately may permit symbiotic advancement of biofilm-forming probiotic *Bacilli* with distinctive lactic acid bacteria (LAB; Kimelman and Shemesh, 2019). Besides, certain vitamins produced by probiotic *Bacilli* can promote cellular function if they survive harsh environmental barriers such as the colon and GIT. For example, LAB can synthesize folate, a B-group vitamin that humans cannot synthesize and must be exogenously obtained (Mosso et al., 2018). Therefore, increasing the folate content of tuber-based foods using LAB may provide novel food matrices to delivery probiotic microorganisms to humans (Mosso et al., 2018). Furthermore, probiotic *Bacilli* may facilitate the production of vitamin B_12_ an essential water-soluble vitamin vital to prevent severe pathologies, some of which are irreversible (Molina et al., 2012).

**FIGURE 2:**
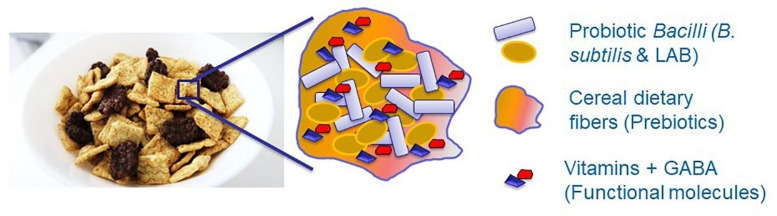
The conceptual idea for developing synbiotic food system through facilitating survivability of probiotic cells and inducing production of health promoting functional molecules. Dietary fibers, for instance originated from cereals, can function as a scaffold for proliferation of probiotic species. Besides, these fibers can serve as a prebiotic substances for growth of probiotic bacteria as well as may facilitate production of health promoting molecules. Overall, it is believed that this symbiotic system will enhance survivability of probiotic bacteria against various environmental stresses.

Although the LAB are among the most prominent probiotic microorganisms (Yang et al., 2015; Yahav et al., 2018), they should be successfully established within the GIT system of the host organism to exert their beneficial effect. Consequently, it was recently proposed using the biofilm-inspired encapsulation of live probiotic cells through facilitating production of protecting extracellular matrix (Yahav et al., 2018; Kimelman and Shemesh, 2019) or by lipid-coated delivery system (Cao et al., 2019). Besides, induced production of different health-promoting molecules, such as vitamins and neuroprotective substances, would facilitate the beneficial effects of the probiotic formulations (Figure 2). Therefore, it is believed that this conceptual idea will provide a basis for the development of a synbiotic food system facilitating the survivability of probiotic cells through inducing the production of health-promoting functional molecules.

## Future Perspectives in Developing Effective Synbiotic Food Incorporating the Probiotic *Bacilli*

One of the potentially manageable foods for developing an effective synbiotic food system can be cereal grains, which offer the positive benefits of combining probiotic species with whole grains that may serve as a staple prebiotic substance. Recent studies indicate that certain probiotic microorganisms contain necessary components to be established as synbiotic food, for instance, cereal-based efficient products (Budhwar et al., 2020). Additionally, the usage of certain microorganisms as beginning cultures throughout the food fermentation process is a notably favorable technique to improve the taste and mineral bioavailability in native cereal-based fermented foods (Nkhata et al., 2018; Ogunremi et al., 2020). Fermented foods are superior in nutrients compared to their unfermented counterparts due to the activation of endogenous enzymes that degrade antinutritional factors. Antioxidant properties of fermented foods are also elevated compared to their unfermented counterparts due to increased vitamin C and ease of release of different health-promoting bioactive compounds resulting from a weakening of grain matrix (Nkhata et al., 2018).

The preparation of cereals with advanced approaches creates an enhanced nutrient platform with a preferred amino acid pattern. Fermentation is considered an essential and accepted method, significantly decreasing the antinutrients existing in coarse cereals such as trypsin inhibitor, phytic acid, and tannins (Budhwar et al., 2020). Phytase activity is a beneficial technological characteristic in LAB proposed to be administered as starters in cereal and legume fermentations. Significant phytase activity in the presence of simulated gastrointestinal (SGI) fluids along with the ability to produce phytases post-exposure to the SGI fluids becomes of high interest (Amritha and Venkateswaran, 2018). Therefore, it augments the full nutritional value of coarse cereals and other food grains.

It is also apparent that different food matrices, for instance, dietary fibers of various food products, might serve as a natural scaffold for probiotic bacteria to adhere to and grow as biofilms. It was lately reported that the probiotic *Bacilli* could interact with resistant starch fibers of chickpea milk (CPM), along with the production of a reddish-pink pigment (Rajasekharan et al., 2020). Interestingly, the probiotic cells could use the resistant starch fibers as a scaffold and modify them to digestible fibers from another side (Rajasekharan et al., 2020). This finding may inspire the use CPM as a dietary supplement enriched with probiotics. CPM could serve as a natural source for prebiotics, the microbiome-shaping components that provide the carbon source for the beneficial microbes in the human gut. These fibers pass through the GIT virtually intact and undigested. In the lower GIT, they are utilized by gut microbiota, which digests them to distribute nutrients to the colonic epithelium, thus maintaining a functional and healthier digestive system (Rhayat et al., 2019). Enriching CPM with probiotics will generate a blend of the synbiotic complex, which might help the probiotic cells during transit through the acidic gastric environment without being killed (considering prebiotics might protect them and allow fast passage through the GIT).

**FIGURE 3:**
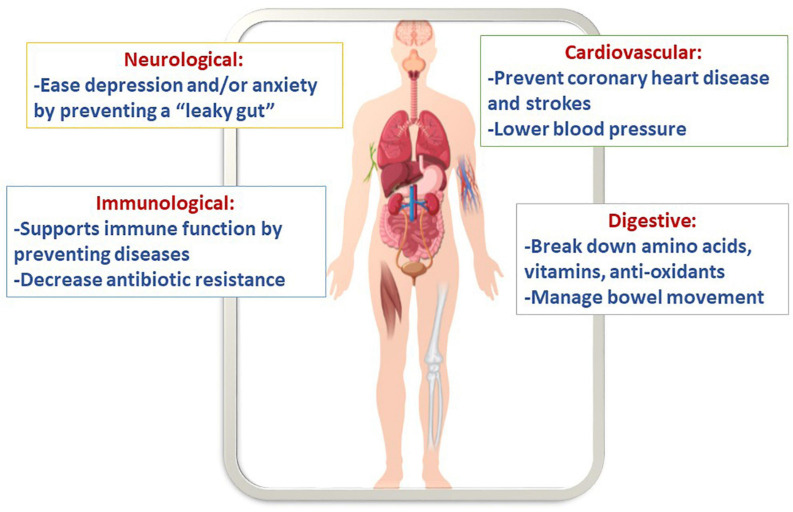
Putative health benefits of synbiotic cereals.

Arguably, synbiotic cereals can promote health because gut microbiota demonstrated to imply a pronounced impact on numerous cellular and host functions (Figure 3). For example, these foods show an improvement in immunology, neurological functions, energy, storage, etc. Several bio-polymers can be hydrolyzed by probiotic bacteria into tiny metabolites that can be used right away. Some of these metabolites include amino acids, essential vitamins, and anti-oxidants, which are produced by the beneficial microbiota strains (Cukkemane et al., 2020). Additionally, it was reported that cereal grains consumption might prevent coronary disease and strokes (Anand et al., 2015). Moreover, probiotics are efficient in managing bowel movement and controlling pathogens such as *Clostridium difficile*, *Campylobacter jejuni*, and *Helicobacter pylori* (Roman et al., 2019). Since cereals are made of grains they contain a staple source of carbohydrate, dietary fiber, and protein. In addition, they are a suitable source of vitamins, such as the B- and E-groups of vitamins, and different vital minerals, such as iron, zinc, magnesium, and phosphorus. Moreover, phytochemicals, including phytoestrogens, antioxidants, and phenolics, are found in whole grain foods. When the phytochemicals are combined with vitamins and minerals, they could defend against gastrointestinal cancers and cardiovascular disease (Flight and Clifton, 2006).

Apparently, biofilm-forming probiotic *Bacilli* incorporated into the synbiotic cereals have a vast potential in survivability during the transition of acidic pH and subsequent establishment in the GIT. Moreover, the health-promoting activity of the cereals may further contribute to strengthening important immune responses to various infectious agents.

## Concluding Remarks

As discussed throughout this communication, prebiotic and synbiotic foods can promote various health aspects in host organisms through different mechanisms. Primarily, the probiotic microbiota should promote the digestion of dietary fibers through enabling the proper functionality of the innate and adaptive immune system of the host organism.

Since Gram positive *Bacilli* are noticeable colonizers of the human GIT tract, they could be used as probiotic species in clinical practices to increase the body’s defense mechanisms against infectious diseases. Predominantly, the normal microbiota contributes to food digestion and the development as well as the optimal functioning of the immune system. Therefore, the probiotic *Bacilli* obtained with food can be beneficial in stimulating a healthiness in human through obtaining bacterial viability in the acidic conditions of the stomach and the high bile concentration in the small intestine. Thus, recently developed biofilm-inspired encapsulation systems may protect probiotic *Bacilli* using food matrices such as dietary fibers. We discussed how certain dietary fibers might serve as the scaffold for the probiotic *Bacilli* to colonize them through forming multicellular communities. The fibers can essentially promote protection by encapsulating probiotic bacteria against various environmental and physical stresses that might kill the free-living bacterial cells. Besides, these fibers would serve as a prebiotic substance that would eventually be utilized by the probiotic cells. Therefore, it is feasible to apply this novel platform for various applications, for instance, probiotic and synbiotic food: snacks, candies, and cereals. Additionally, the synbiotic food harboring probiotic species can antagonize pathogenic bacteria, involved in different diseases from dental caries to irritated bowel syndrome.

## Author Contributions

Both authors developed a conceptual idea, originated the draft, prepared the illustrations for the manuscript, discussed and elaborated on the role of probiotic *Bacilli* in human health, and approved the final version of the manuscript.

## Conflict of Interest

The authors declare that the research was conducted in the absence of any commercial or financial relationships that could be construed as a potential conflict of interest.
